# A Rare Cause of Acute Pancreatitis: Intramural Duodenal Hematoma

**DOI:** 10.1155/2012/275604

**Published:** 2012-10-04

**Authors:** Hemant Goyal, Umesh Singla, Roli R. Agrawal

**Affiliations:** Department of Internal Medicine, Wyckoff Heights Medical Center, Brooklyn, NY 11237, USA

## Abstract

We describe an interesting case of intramural duodenal hematoma in an otherwise healthy male who presented to emergency room with gradually progressive abdominal pain, nausea, and vomiting. This condition was missed on initial evaluation and patient was discharged from emergency room with diagnosis of acute gastritis. After 3 days, patient came back to emergency room and abdominal imaging studies were conducted which showed that patient had intramural duodenal hematoma associated with gastric outlet obstruction and pancreatitis. Hematoma was the cause of acute pancreatitis as pancreatic enzymes levels were normal at the time of first presentation, but later as the hematoma grew in size, it caused compression of pancreas and subsequent elevation of pancreatic enzymes. We experienced a case of pancreatitis which was caused by intramural duodenal hematoma. This case was missed on initial evaluation. We suggest that physicians should be more vigilant about this condition.

## 1. Case Report

A 29-year-old male patient with no significant medical or surgical past history presented to our emergency room (ER) with complaints of gradually progressive abdominal pain of two-week duration associated with recent development of nausea and vomiting. Abdominal pain was located in epigastrium, intermittent, 6/10 in severity, nonradiating, and was dull in nature. Patient also stated that he took some unknown over-the-counter antacid to relieve the pain but it did not help. Patient also complained of nausea and vomiting which started after his moderate alcohol intake 2 days ago. Patient denied taking any other medications. He also denied any history of trauma. Physical examination was negative except for presence of tenderness in epigastric area. Laboratory exam was completely normal including amylase and lipase levels (Table 1). A diagnosis of alcoholic gastritis was made and patient was discharged from ER with a proton pump inhibitor.

This patient again came to ER after about 72 hours with complaints of increased abdominal pain and intractable nausea and vomiting. On examination patient had generalized abdominal fullness and tenderness. Bowel sounds were sluggish on auscultation. Rectal examination was normal with stool negative for occult blood. Laboratory examination was abnormal with elevated levels of amylase and lipase. Laboratory examinations for day 1 and day 2 of presentation to ER are elaborated in Table 1.

**Figure 1:**
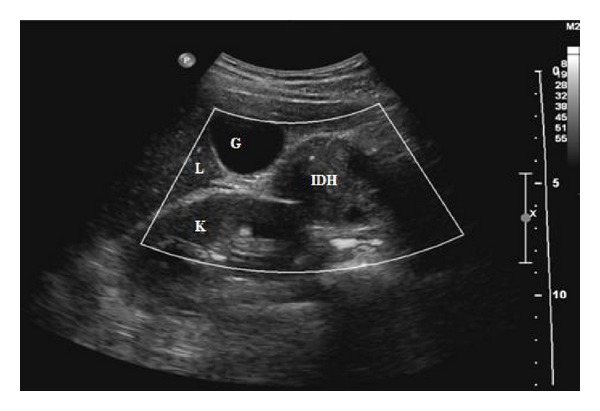
Ultrasound of the abdomen demonstrates a well-defined heterogenous 3.5 × 3 cm lesion anterior to the right kidney in the location of second part of duodenum. IDH: intramural duodenal hematoma; L: liver; G: gall bladder; K: kidney.

**Figure 2:**
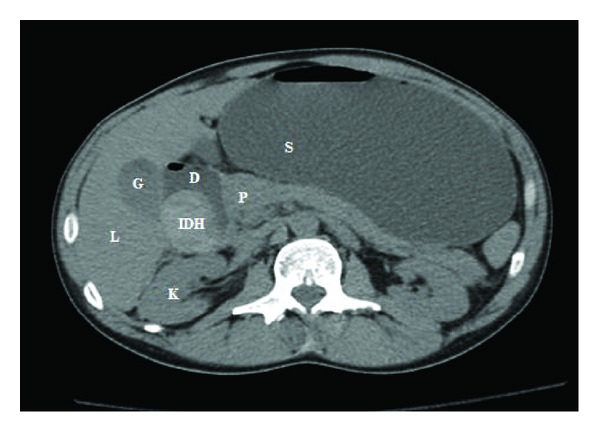
Axial image of noncontrast CT scan of the abdomen at the level of second part of duodenum demonstrates a circumferential hyperdense lesion in the wall of the second part of duodenum with peripancreatic fat stranding. IDH: intramural duodenal hematoma; D: second part of duodenum; P: pancreas; S: stomach; L: liver; K: kidney; G: gall bladder.

Ultrasonogram of the abdomen demonstrated a 3.5 × 3 cm heterogenous, well-defined lesion anterior to the right kidney in the location of second part of duodenum (Figure 1). This lesion failed to show any vascularity on Doppler sonogram. Computed tomography (CT) scan of abdomen without intravenous (IV) contrast showed a circumferential hyperdense lesion in the wall of the second part of duodenum with no definite mass with presence of minimal peripancreatic fat stranding. There was associated gastric outlet obstruction (Figure 2). CT scan with IV contrast could not be performed as patient had acute renal failure at the time of presentation.

**Figure 3:**
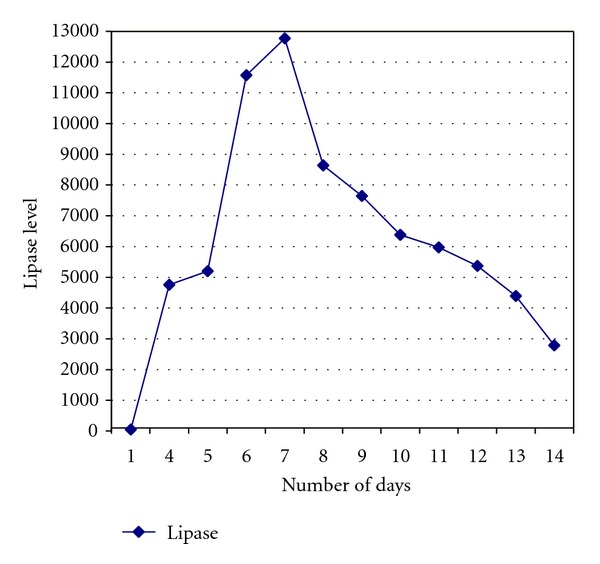
Pancreatic lipase levels of the patient which started to decline when hematoma started to resolve.

Patient was started on nil per oral and aggressive hydration therapy. Continuous gastric suction was performed by using a nasogastric tube. Laboratory blood tests were monitored at regular intervals and significant elevations in lipase, amylase, BUN, creatinine, and WBC were observed initially which started to trend down later. A graph depicting the levels of lipase is shown (Figure 3). Serial abdominal CT scans without IV contrast were done (Total 3) and patient was managed symptomatically. Patient's hematoma slowly started to regress and pancreatic enzymes levels came down. Patient was admitted in the hospital for total of 12 days. His hematoma completely resolved at the time of discharge and he was completely asymptomatic.

## 2. Discussion

IDH is usually seen in children after blunt abdominal trauma, though this condition has also been rarely reported in adults. In adults this condition has been described in patients who had risk factors like history of abdominal trauma [1], aspirin [2], or warfarin [3] intake, had undergone some endoscopic intervention [4] or in association with pancreatitis [5]. In our case we could not find the cause of IDH as patient did not have bleeding disorder or other risk factors for IDH. Our patient had normal levels of pancreatic enzymes at the time of first presentation, therefore we believe that he did not have pancreatitis at that time. Later due to pancreatic compression by IDH, patient developed pancreatitis. Similarly, CT scan of abdomen was suggestive of mild pancreatitis which was not severe enough to cause IDH in our patient as described previously in literature.

Relative fixed position of duodenum and its proximity to vertebral column along with rich vascular supply is considered to be the culprit for IDH. The most common presenting symptoms are abdominal pain, nausea, and vomiting. Laboratory tests are of limited value in diagnosis of IDH associated with pancreatitis especially in early course of the disease. Patient can develop anemia due to loss of blood in hematoma. Pancreatic compression due to IDH can cause elevated pancreatic enzymes levels [6]. In our case we believe that IDH was the cause of acute pancreatitis as pancreatic enzymes levels were normal at the time of first presentation, but later as the hematoma grew in size, it caused compression of pancreas and subsequent elevation of pancreatic enzymes. Diagnosis of this condition relies on clinical suspicion and radiological studies. Contrast-enhanced computed tomography scan is preferred over magnetic resonance imaging because of its availability and cost effectiveness. In our case contrast-enhanced CT scan was not performed because of elevated creatinine. Most uncomplicated IDH resolve spontaneously. Surgical intervention is reserved for the patients in whom conservative treatment fails or complications like perforation or bowel infarction occur. This is why close monitoring of these patients is very crucial. In this era of minimally invasive procedures ultrasound or CT guided drainage of IDH is preferred over laparotomy. Recently laparoscopic drainage of IDH has also been successfully performed [7]. Our patient did not undergo any surgical intervention because patient was hemodynamically stable and was responding well to conservative treatment.

IDH is a very rare diagnosis but it is crucial to diagnose early as most of the patients can be treated conservatively. IDH is a diagnosis of clinical suspicion and confirmation can only be done with abdominal imaging studies.

## Figures and Tables

**Table 1:** Comparison of laboratory examinations of patient for day 1 and day 2 of presentations in emergency room.

Lab value	Day 1	Day 2
Calcium	10.6 mg/dL	8.7 mg/dL
Blood urea nitrogen	9 mg/dL	141 mg/dL
Creatinine	0.8 mg/dL	9.2 mg/dL
Lipase	50 units/L	5036 units/L
Total bilirubin	1.2 mg/dL	1.6 mg/dL
Direct bilirubin	<0.1 mg/dL	0.1 mg/dL
ALT	27 units/L	30 units/L
AST	49 units/L	97 units/L
Alkaline phosphatase	130 units/L	Not Performed
White blood cells (WBCs)	9800/mm^3^	19800/mm^3^
Hemoglobin	14.5 gm/dL	13.4 gm/dL
Hematocrit	40.9%	38.9%
INR	1.03	0.95
Aptt	27.2 seconds	24.8 seconds
PT	11 seconds	10.2 seconds
